# Social ecological factors associated with experiencing violence among urban refugee and displaced adolescent girls and young women in informal settlements in Kampala, Uganda: a cross-sectional study

**DOI:** 10.1186/s13031-019-0242-9

**Published:** 2019-12-17

**Authors:** Carmen H. Logie, Moses Okumu, Simon Mwima, Robert Hakiza, Kibathi Peter Irungi, Peter Kyambadde, Emmanuel Kironde, Manjulaa Narasimhan

**Affiliations:** 1 Factor Inwentash Faculty of Social Work, University of Toronto, 246 Bloor Street West, Toronto, ON M5S 1V4 Canada; 2 Women’s College Research Institute, Women’s College Hospital, 76 Grenville St, Toronto, ON M5G 1N8 Canada; 3 School of Social Work, University of North Carolina-Chapel Hill, 325 Pittsboro ST CB#3550, Chapel Hill, NC 27599 USA; 4 National STI Control Unit, Mulago Hospital, Kampala, Uganda; 5 AIDS Control Program, Ministry of Health, Plot 6, Lourdel Road, Nakasero, Kampala, Uganda; 6 Most At Risk Population Initiative, Kampala, Uganda; 7 Young African Refugees for Integral Development (YARID), Nsambya Gogonya, Kampala, Uganda; 8 Tomorrow Vijana, Plot 20 Hanlon Road, Nsambya, Kampala, Uganda; 9 Interaid Uganda, Kabaka Anjangala Road, Kampala, Uganda; 10 Department of Reproductive Health and Research, World Health Organization, 20 Avenue Appia, CH-1211, 27 Geneva, Switzerland; 11 World Health Organization, Department of Reproductive Health and Research, including the UNDP/UNFPA/UNICEF/WHO/World Bank Special Programme (HRP), Geneva, Switzerland

**Keywords:** Refugee, Displaced, Adolescent girls and young women, Violence, Polyvictimization, Uganda, Urban, Survivors, Social context, Relationship power

## Abstract

**Background:**

Research on violence targeting urban forcibly displaced adolescent girls and young women (AGYW) is limited, particularly regarding polyvictimization (exposure to multiple forms of violence). Yet there is a global trend of refugee urbanization, and urban AGYW are at the nexus of violence disparities among adolescents, forcibly displaced persons, and slum dwellers. This study explored factors associated with young adulthood violence (> 16 years) (YAV) and intimate partner violence (IPV) among forcibly displaced AGYW in Kampala, Uganda.

**Methods:**

We conducted a cross-sectional survey with forcibly displaced AGYW aged 16–24 from five informal settlement (slum) communities across Kampala (Kabalagala, Rubaga, Kansanga, Katwe, Nsambya) using peer network sampling. We assessed YAV (experienced at age 16 or above) (sexual, physical, emotional violence) and recent (past 12-month) IPV (physical, sexual, control violence). We conducted descriptive statistics, followed by multinomial logistic regression analyses to explore social ecological factors (e.g., *intrapersonal:* depression; *interpersonal:* sexual relationship power, *community:* food insecurity) associated with experiencing YAV and YAV polyvictimization, and IPV and IPV polyvictimization.

**Results:**

Over half of participants (*n* = 333; mean age = 19.31; SD = 2.56, range = 16–24) reported YAV (*n* = 179; 53.7%) and 9.3% (*n* = 41) reported YAV polyvictimization. Most participants that were in an intimate relationship in the last 12 months (*n* = 200; 85.8%) reported IPV, among these, 45.5% reported one form of IPV and 54.5% reported IPV polyvictimization. In adjusted analyses, experiencing any YAV was significantly associated with: adolescent sexual and reproductive health (SRH) stigma; sexual relationship power; mobile app usage; depressive symptoms; childhood abuse; and childhood polyvictimization. In adjusted analyses YAV polyvictimization was associated with: depressive symptoms; childhood polyvictimization; sexual relationship power; and food insecurity. Recent IPV polyvictimization in adjusted analyses was associated with owning/using a mobile phone and depressive symptoms. Participants with higher sexual relationship power had lower odds of recent IPV polyvictimization.

**Conclusion:**

Findings suggest that YAV and IPV polyvictimization require urgent attention among forcibly displaced AGYW in Kampala. Multi-level strategies are required to address *intrapersonal* e.g. (depression), *interpersonal* (e.g. childhood abuse, sexual relationship power) and *community* (e.g. adolescent SRH stigma, food insecurity) factors associated with experiencing violence. Future research can tailor approaches to advance health, agency and human rights among urban forcibly displaced AGYW.

## Background

Sexual and gender-based violence (SGBV) is a human rights violation and public health priority [1, 2] that disproportionately impacts women and children in humanitarian settings [3, 4]. An estimated one-fifth—21.4% —of refugee women have reported experiencing sexual violence [4]. A confluence of factors contributes to increased community and intimate partner violence exposure in humanitarian settings, including family and community network breakdown that reduces social support access, increased poverty and food insecurity, and changes in family dynamics and gender roles [4–7]. SGBV is also used systematically as a weapon of war [8]. The ways in which SGBV is enacted during conflict thus spans social ecological levels across micro (intimate partnerships), meso (community norms), and macro (military actors) levels [8, 9]. Sequalae of experiencing SGBV include poorer physical, reproductive and mental health that reduce quality of life across the life course [10–12].

Sexual and gender-based violence is a global concern with varied prevalence across contexts and studies. Within sub-Saharan Africa, IPV prevalence differs in conflict situations. For instance, in stable countries such as Tanzania, Vyas & Heise [12] reported that 36% of women in intimate relationships experienced IPV. Yet in conflict affected countries such as Democratic Republic of Congo (DRC), Tlapek [13] reported an IPV prevalence nearly two-fold higher—68%. Similarly, a study including children and their women guardians in conflict affected Northern Uganda reported an IPV prevalence among women of 52% compared to 15% in other stable regions of the country [14]. Conflict-affected adolescent girls aged 15–22 in South Sudan reported high lifetime experiences of non-partner sexual violence (26.5%) and lifetime intimate partner violence (43.1%) [15]. A 2019 systematic review by Seddighi and Colleagues [16] called for more evidence regarding SGBV experienced by adolescent girls and young women (AGYW) to inform the development of effective prevention and response interventions in humanitarian settings.

Uganda is an important case study to examine SGBV as a low-income country and Africa’s largest refugee hosting nation with over 1.3 million refugees [9, 17]. A recent national study on violence experiences among 13–24 year-old Ugandans reported that 35% experienced sexual violence, 59% physical violence, and 33.3% reported experiencing emotional violence [18]. Scant evidence exists on the nature, extent and prevalence of SGBV among forcibly displaced persons in Uganda [19]. The term forcibly displaced refers to: internally displaced persons who left their home but did not cross an international border; refugees who left their home country due to conflict, war and/or violence; and asylum seekers who left their home country and applied for sanctuary in a different country [17]. In a multi-country cross-sectional study including 1296 conflict-affected adolescent girls from DRC, South Sudan, and Ethiopia, Stark and colleagues [20] reported that over half of participants experienced at least one type of violence in the previous 12 months, including: 31.8% physical violence; 36.8% emotional violence; and 26.7% sexual violence.

The 2006 Uganda Refugee Law supports forcibly displaced persons to leave refugee settlements and camp settings, with most resettling in informal urban settlements (also referred to as “slums”). Informal settlements often have higher incidences of violence due to economic insecurities that can result in familial conflict and insecure living situations [21]. For instance, a study examining the prevalence and correlates of violence against AGYW aged 14–24 in Kampala’s informal settlements (*n* = 313) reported that 37% of youth engaged in physical fights, 28% were threatened or injured with a weapon, and 30% experienced forced sex [22]. With most (56%) of sub-Saharan Africa’s urban population living in informal settlements [23], and approximately 100,000 refugees living in Kampala’s informal settlements [19], there is an urgent need to understand and address SGBV among forcibly displaced AGYW in these settings.

Urban forcibly displaced AGYW are at the nexus of violence targeting refugees and violence in informal settlements, yet little is known of their experiences of violence. Research with refugees has traditionally focused on sexual violence in settlement/camp settings [4], overshadowing the urbanization of refugees. Polyvictimization—cumulative and multiple forms of violence spanning familial, community and intimate partners, among others—is understudied among forcibly displaced AGYW. Yet among AGYW who have experienced violence, most have experienced polyvictimization [24] and polyvictimization contributes to more variability in mental health outcomes in comparison with individual forms of violence [25].

A 2017 [26] systematic review including 32 cross-sectional studies in humanitarian settings identified that exposure to violence against women and violence against children was associated with: low income, alcohol and drug use, mental health and coping strategies, and low social support. Economic insecurity exacerbates risks of violence among refugee and displaced AGYW in complex ways. Forcibly displaced AGYW may engage in transactional sex to support individual and family survival [27, 28], in turn, transactional sex may elevate exposure to violence [29, 30]. Economic insecurity may challenge men’s traditional expectations of gendered roles as the breadwinner, in turn contributing to IPV. For instance, young women in post-earthquake Haiti contextualized men’s violence in relationship to frustration regarding food and housing insecurity and a lack of employment options [27]. A qualitative study in post-conflict Colombia [31] applied a social ecological lens to understand the interconnections between community violence, violence against children, and IPV. This study reported migration stressors when moving from rural to urban settings, including economic challenges acquiring food, housing and employment as well as intensified violence within families [31].

Other community factors such as inequitable gender norms may be associated with violence [32] targeting women. For instance in South Sudan gender inequitable norms were associated with acceptance of violence toward women [33]. Stigma directed toward adolescent sexual practices and engagement in sexual and reproductive health (SRH) services, such as contraception and HIV testing, is also associated with social isolation, violence, and mental health challenges [34, 35]. Associations between adolescent SRH stigma and violence is understudied among refugee and displaced AGYW.

At the individual/intrapersonal level, mental health challenges such as depression may have a reciprocal relationship with violence [36]. Experiencing violence is associated with increased likelihood of experiencing depression and post-traumatic stress disorder [36, 37], and depressive symptoms may constrain self-efficacy, optimism and motivation needed to acquire support and resources needed to leave violent situations [38]. A cross-sectional study with adolescent refugees from the DRC living in Rwanda and from South Sudan living in Uganda found that individuals with mental health challenges were more likely to report experiencing violence [39]. In Uganda, mobile phone and internet use has increased exponentially from 40,000 in 2000 to 19,000,000 in 2016 [40]. Mobile phone and internet use are a lifeline for refugees as they communicate, seek services, and navigate new environments [41]. However, mobile phones and the internet can also be used to perpetuate and exacerbate SGBV targeting women and girls [29]. ‘Technology-facilitated GBV’ includes harm caused using the internet, mobile technology, and comprises stalking, sexual harassment and other forms of exploitation [42, 43]. Specifically, digital technologies may result in concerns and blame regarding infidelity, in turn contributing to partner violence. A 2018 systematic review [43] called for studies conducted in low-and middle-income countries to investigate the impact of digital technologies on SGBV. A 2019 commentary [45] argued that digital technologies have a potential role to play in SGBV prevention, yet researchers should also consider “the potential for harm with web-based technologies in violent relationships, in which possessive partners might search mobile phones” (pp e270). This suggests that a deeper understanding is needed regarding the role of digital technologies in both reducing and increasing risks for SGBV.

The experiences of violence among urban refugee and displaced AGYW remain understudied across global contexts, including Uganda. To address this knowledge gap, this study explored experiences of violence from any person, as well as from intimate partners. Specifically we examined: (1) the prevalence of experiencing violence (emotional, physical, sexual) in young adulthood (during or after the age of 16) the prevalence of experiencing recent (past 12 month) intimate partner violence (IPV); and (3) socio-demographic and social ecological factors associated with young adulthood violence and recent IPV at *intrapersonal* (mobile phone engagement, depression), *interpersonal* (childhood violence, transactional sex, sexual relationship power), and *community* (adolescent sexual and reproductive health stigma, food insecurity, community safety) levels among urban refugee and displaced AGYW living in Kampala’s informal settlements.

## Methods

### Participants

We conducted a cross-sectional survey as part of a larger HIV prevention-focused community-based research study with young women (16–24 years) refugees living in informal settlements of Kampala. Participants completed an interview-administered questionnaire between January to March 2018. Data were collected in collaboration with refugee agencies (Interaid Uganda, Young Africans for Integral Development (YARID), Tomorrow Vijana) and government agencies (Uganda AIDS Control Program, Ministry of Health). Inclusion criteria included: adolescent girls and young women aged 16–24; self-identified as a refugee or displaced person or having refugee/displaced parents; living in one of 5 informal settlements (“slums”) in Kampala (Kabalagala, Rubaga, Kansanga, Katwe or Nsambya); and able to provide informed consent. Congruent with recommendations for ethical approaches to adolescent participation in sexual health research [46], we did not require parental consent for participation for those aged 16–17 years for the following reasons: 1) adolescents in Uganda are able to receive an HIV test after the age of 12 years independently, and participants were able to understand this study given their exposure to multi-media and community-based campaigns to end violence in Uganda, 2) seeking parental consent would have prevented some participants from freely discussing their experiences, and 3) we received a waiver from the ethics boards.

### Recruitment

We recruited and trained 8 peer research assistants (PRAs) including women from Burundi (*n* = 2), the Democratic Republic of Congo (*n* = 4), South Sudan (*n* = 1) and Rwanda (n = 1) who self-identified as refugee or displaced girls/women aged 18–24 to recruit participants and to administer the tablet-based survey. We used peer-driven and peer network sampling methods to recruit participants for the study. Peer network sampling [48] is an effective strategy to recruit and include hidden and marginalized populations such as urban refugees and displaced youth in research. Participants were given study ‘coupons’ and encouraged to recruit between one to five participants from their social networks. Subsequent participants were invited to recruit 2–5 persons from their social networks until the target number of participants was reached. A trained social worker provided psychosocial support to participants in cases of distress (no cases were reported). All participants received a handout with psychosocial resources and the PRAs provided further information regarding resources in Kampala for violence prevention and response, including information on mental health support services (for instance, at Interaid Uganda) and access to post-exposure prophylaxis. The study received research ethics approval from the University of Toronto, Canada and the Ugandan Ministry of Health.

### Data collection procedures

Prior to beginning the study, all participants provided informed consent on the tablet. The PRAs administered the tablet-based surveys in English, French or Swahili, at a preferred location chosen by the participants. Participants received an honorarium of UGX 12,500-shilling (∼$3.72 USD) for completing a 35–45 min survey.

### Measures

#### Outcome variables: lifetime experiences of young adulthood violence and recent intimate partner violence

We assessed (1) violence during and/or after the age of 16 years (lifetime experiences of young adulthood violence [YAV]) and (2) intimate partner violence in the last 12 months (recent IPV). Violence included three types of violence: having experienced physical, verbal, and/or sexual violence. To assess young adulthood violence, participants were asked: *Have you ever experienced at age 16 years old and over (check all that apply); sexual violence (yes = 1, no = 0); physical violence (yes = 1, no = 0) or verbal abuse (yes = 1, no = 0).* The IPV questions were completed by participants who were currently or were in an intimate relationship in the past 12 months. Questions assessed physical, sexual and control violence in the last 12 months. For instance, physical violence was assessed with the question: “*During the past 12 months how many times did someone you were dating physically hurt you on purpose (such as hit, slammed you into something)*; sexual violence was assessed with the question, *during the past 12 months how many times did someone you were dating force you to do sexual things you did not want to do (such as kissing, sex)*; and control violence was assessed with the question, “*during the past 12 months how many times did someone you were dating try to control your actions (e.g. who you hang out with, where you are)*.” Response options included: Never, 1 time, 2 or 3 times, 4 or 5 times, and 6 or more times. For lifetime young adulthood violence, and for recent IPV, responses were categorized into: no violence = 0; experienced 1 type of violence = 1; and experienced more than one type of violence (polyvictimization) = 2.

***Socio-demographic*** variables included age (continuous), education level (categorical: no education/less than secondary school, and post-secondary education), employment status (categorical: employed, unemployed, and student), and relationship status (categorical: no relationship, dating one partner/married and casual dating/multiple partners).

***Intrapersonal level factors*** included mobile phone ownership and use, mobile application (app) usage, and depression. *Mobile phone ownership and use* was assessed by asking if participants owned and used a mobile phone. An affirmative response was coded as *mobile phone ownership and use*. *Mobile app usage* was measured categorically by asking participants to indicate the apps (e.g. WhatsApp, Viber, Facebook, Snapchat and Telegram) used to communicate with friends and others. Responses to this item were recoded into three categories: No app = 0; one app = 1; and more than one app = 2. *Depression* was assessed using the Patient Health Questionnaire-9 (PHQ-9) [47]. Before categorizing the instrument, we found acceptable internal reliability of the PHQ-9 among this sample of refugee and displaced AGWY in Kampala (Cronbach Alpha = 0.89). Following scoring guidelines [47], participants who had scores less than 5 were classified as having no depressive symptoms, while participants with a score of 5 or above were classified as having depressive symptoms.

***Interpersonal level factors*** included transactional sex and experiencing childhood abuse. *Transactional sex* was assessed by asking participants if they had exchanged sex in their lifetime for: money; drugs; shelter; food; gifts; clothes; services; and/or other reasons. An affirmative response was coded as transactional sex engagement. *Experiencing childhood abuse* was assessed by asking: *Have you ever experienced as a child (below the age of 16) (check all that apply); sexual violence (yes = 1, no = 0); physical violence (yes = 1, no = 0) or verbal abuse (yes = 1, no = 0).* Responses were categorized into: No childhood violence = 0; experienced 1 type of childhood violence = 1; and experienced more than one type of childhood violence = 2. *Sexual relationship power* [48] was assessed using the 15-item relationship control subscale from the Sexual and Relationship Power Scale (SRPS) using a 4-point Likert scale (1 = strongly agree to 4 = strongly disagree). The relationship control subscale assesses power and control in sexual relationships (e.g. *If I asked my partner to use a condom, he would get violent*). We calculated scores using SRPS testing guidance [48]. The scale had acceptable reliability (Cronbach Alpha = 0.90) within our sample. Higher SRPS scores indicated higher sexual relationship power.

***Community level factors*** included adolescent sexual and reproductive health (SRH) stigma, food insecurity and community safety perceptions. We used the 19-item Adolescent SRH Stigma Scale (Cronbach’s α = 0.85, range 0–19 in our study) [49] to measure *adolescent sexual and reproductive health (SRH) stigma* validated among this sample of urban refugee and displaced AGYW [50]. The scale is used to assess stigmatizing attitudes toward adolescents’ involvement in sex, pregnancy, abortion, and accessing STI services and family planning [49]. Response options used a 3-point Likert scale (disagree, neutral, agree) and were summed to create a total stigma score. *Food insecurity* was assessed using the question: “*how often do you go to bed hungry because you didn’t have enough to eat*?” with a 4-point Likert scale (never, sometimes, most days, everyday). We dichotomized responses into yes = 1—experienced food insecurity and No = 0—did not experience food insecurity [51]. *Community safety* perceptions were assessed using the item: *“how safe do you feel in terms of your physical safety in your community (e.g. crime, violence)?”* This variable included 4-response options (not safe, fairly safe, safe, very safe) that we dichotomized into yes = 1—safe and No = 0—not safe.

### Data analysis

We calculated summary statistics of socio-demographic, SGBV, and social ecological variables, using means and standard deviation (SD) for continuous variables, and frequencies and proportions for categorical variables. We examined the data using one-way analysis of variance (ANOVA) for continuous variables and Chi-square or Fisher’s Exact test for categorical variables to assess differences in young adulthood violence (YAV) and recent IPV experiences across socio-demographic and social ecological variables. Unadjusted and adjusted multinomial logistic regression (MLR) analyses were conducted to identify social ecological factors associated with 1) lifetime YAV, 2) YAV polyvictimization, 3) recent IPV, and 4) IPV polyvictimization. We adjusted for variables that were significant in the unadjusted models.

## Results

### Prevalence of young adulthood violence and recent intimate partner violence

There were 333 participants (mean age = 19.31; SD = 2.56, range = 16–24). Tables 1 and 2 summarize participants’ socio-demographic information and associations with experiencing one type of violence and polyvictimization (> 1 type of violence). The mean participant age for participants (*n* = 333) who responded to lifetime YAV questions was 19.31 years (SD = 2.56), and the mean age of participants (*n* = 233) who completed the recent IPV survey was 20.01 years (SD = 2.48).

**Table 1:** Socio-demographic factors and violence among refugee/displaced adolescent girls and young women in Kampala, Uganda (*N* = 333)

Sociodemographic factor	Total *N*	Overall (*N* = 333)		No violence reported (*N* = 154, 46.2%)		1 type of violence reported (*N* = 148, 44.4%)		+1 type of violence reported (*N* = 31, 9.3%)		*P* value
		*N*	(%)	*N*	(%)	*N*	(%)	*N*	(%)	
Age at interview date (years)	333	Mean = 19.31, SD = 2.56	Range = 16–24	Mean = 18.84, SD = 2.36	Range = 16–24	Mean = 19.60, SD = 2.64	Range = 16–24	Mean = 20.23, SD = 2.75	Range = 16–24	< 0.004
Education	333									< 0.355
Less than post-secondary school		190	(57.1)	93	(60.4)	78	(52.7)	19	(61.3)	
Post-secondary education		143	(42.9)	61	(39.6)	70	(47.3)	12	(38.7)	
Country of birth	333									< 0.034
South Sudan		30	(9.0)	5	(3.2)	20	(13.5)	5	(16.1)	
Burundi		111	(33.3)	51	(33.1)	53	(35.8)	7	(22.6)	
DR Congo		153	(45.9)	80	(51.9)	58	(39.2)	15	(48.4)	
Rwanda		19	(5.7)	8	(5.2)	10	(6.8)	1	(3.2)	
Others		20	(6.0)	10	(6.5)	7	(4.7)	3	(9.7)	
Immigration status	330									< 0.325
Refugees		305	(92.4)	136	(90.1)	140	(94.6)	29	(93.5)	
Asylum seeker/undocumented		25	(7.6)	15	(9.9)	8	(5.4)	2	(6.5)	
Time in Uganda 333										< 0.106
>1 year		209	(62.8)	15	(9.7)	6	(4.1)	2	(6.5)	
1–5 years		84	(25.2)	87	(56.5)	78	(52.7)	21	(67.7)	
<5 years		40	(12.0)	52	(33.8)	64	(43.2)	8	(25.8)	
Employment	333									0.000
Students		155	(46.5)	71	(46.1)	73	(49.3)	11	(35.5)	
Employed		46	(13.8)	9	(5.8)	34	(23.0)	3	(9.7)	
Unemployed		132	(39.6)	74	(48.1)	41	(27.7)	17	(54.8)	
Relationship status	312									< 0.071
No current partner		144	(46.2)	76	(54.7)	57	(39.9)	11	(36.7)	
Dating one partner/married		131	(42.0)	52	(37.4)	65	(45.5)	14	(46.7)	
Casual dating/multiple partners		37	(11.9)	11	(7.9)	21	(14.7)	5	(16.7)	
Mobile phone ownership and use	333									0.043
No		95	28.53	53	(34.4)	32	(21.6)	10	(32.3)	
Yes		238	71.47	101	(65.6)	116	(78.4)	21	(67.7)	
Mobile App usage	333									0.000
No		76	22.82	46	(29.9)	20	(13.5)	10	(32.3)	
> 1		52	15.62	32	(20.8)	19	(12.8)	1	(3.2)	
+ 1		205	61.56	76	(49.4)	109	(73.6)	20	(64.5)	
Depressive symptoms	333									0.000
No		87	26.13	56	(36.4)	28	(18.9)	3	(9.7)	
Yes		246	73.87	98	(63.6)	120	(81.1)	28	(90.3)	
Transactional sex engagement	324									0.000
No		238	(73.5)	123	(82.0)	102	(70.8)	13	(43.3)	
Yes		86	(26.5)	27	(18.0)	42	(29.2)	17	(56.7)	
Sexual relationship power	248	Mean = 30.27, SD = 6.40	Range = 11–44	Mean = 29.16, SD = 5.04	Range = 11–44	Mean = 31.19, SD = 7.34	Range = 11–44	Mean = 30.57, SD = 6.26	Range = 13–44	0.061
Adolescent sexual and reproductive health stigma	333	Mean = 13.92, SD = 4.08	Range = 1–19	Mean = 13.13, SD = 4.51	Range = 0–19	Mean = 14.49, SD = 3.35	Range = 5–19	Mean = 15.13, SD = 4.40	Range = 0–19	0.003
Food insecurity	333									0.006
No		95	(28.5)	42	(27.3)	51	(34.5)	2	(6.5)	
Yes		238	(71.5)	112	(72.7)	97	(65.5)	29	(93.5)	
Perceived community safety	333									0.544
Not safe		143	(42.9)	63	(40.9)	64	(43.2)	16	(51.6)	
Safe		190	(57.1)	91	(59.1)	84	(56.8)	15	(48.4)	

Note: 1. *P*-values were calculated using ANOVA or Chi square test

*Types of violence included verbal, physical, and sexual violence

Over half (*n* = 179; 53.7%) of participants reported YAV (physical: *n* = 50, 15%; sexual: *n* = 41, 12.3%; emotional: *n* = 152, 45.6%). Of these, 44.4% (*n* = 148) reported experiencing one type of violence and 9.3% (n = 41) reported >1 type of violence (polyvictimization) (Table 1). Table 2 reveals that most participants who were in an intimate relationship in the last 12 months (85.8%, *n* = 200) reported experiencing IPV (physical violence: *n* = 63, 27%; sexual violence: *n* = 113, 48.5%; control: *n* = 175, 75.1%). Among the 200 participants who reported recent IPV, 45.5% (*n* = 91) reported one type of IPV and 54.5% (*n* = 109) reported IPV polyvictimization.

### Differences in experiences of young adulthood violence

Table 1 presents the findings from bivariate analyses (chi-square independent tests and one-way ANOVA) examining YAV. Young adulthood polyvictimization was higher for: older participants; person who were unemployed (vs student and employed); participants that used/owned mobile phones vs. those with no phone; those who used multiple mobile apps vs. no apps; persons with depressive symptoms vs. no depressive symptoms; lifetime transactional sex engagement; and persons with higher adolescent SRH stigma scores.

### Differences in recent IPV experiences

Table 2 illustrates the results from chi-square independent tests and one-way ANOVA examining differences associated with experiencing IPV in the last 12 months. Recent IPV polyvictimization was associated with: older age; less than post-secondary school education vs. post-secondary education; place of birth as Burundi vs. South Sudan, Rwanda and other locations; dating one partner/married vs. no current partners or casual dating/multiple partners; lifetime transactional sex engagement; and lower sexual relationship power.

**Table 2:** Past 12-month intimate partner violence among refugee/displaced adolescent girls and young women in Kampala (N = 233)

Sociodemographic factor	Total *N*	Overall (*N* = 233)		No violence reported (*N* = 33, 14.2%)		1 type of violence reported (*N* = 91, 39.1%)		+ 1 type of violence reported (*N* = 109, 46.8%)		*P* value
		*N*	(%)	*N*	(%)	*N*	(%)	*N*	(%)	
Age at interview date (years)	233	Mean = 20.01, SD = 2.48	Range = 16–24	Mean = 20.36, SD = 2.48	Range = 16–24	Mean = 19.47, SD = 2.35	Range = 16–24	Mean = 20.35, SD = 2.54	Range = 16–24	< 0.030
Education	233									< 0.016
Less than post-secondary school		116	(49.8)	14	(42.4)	56	(61.8)	46	(42.7)	
Post-secondary education		117	(50.2)	19	(57.6)	35	(38.5)	63	(57.8)	
Country of birth	233									< 0.000
South Sudan		23	(9.9)	4	(12.1)	13	(14.3)	6	(5.5)	
Burundi		83	(35.8)	17	(51.5)	11	(12.1)	55	(50.5)	
DR Congo		98	(42.1)	6	(18.2)	56	(61.5)	36	(33.0)	
Rwanda		17	(7.3)	4	(12.1)	4	(4.4)	9	(8.3)	
Others		12	(5.2)	2	(6.1)	7	(7.7)	3	(2.8)	
Immigration status	231									< 0.130
Refugees		213	(92.2)	29	(87.9)	86	(96.6)	98	(89.9)	
Asylum seeker/undocumented		18	(7.8)	4	(12.1)	3	(3.4)	11	(10.1)	
Time in Uganda 233										< 0.031
> 1 year		15	(6.4)	3	(9.1)	2	(2.2)	10	(9.2)	
1–5 years		135	(57.9)	15	(45.5)	50	(54.9)	70	(64.2)	
> 5 years		83	(35.6)	15	(45.5)	39	(42.9)	29	(26.6)	
Employment	233									0.000
Students		93	(39.9)	18	(54.5)	46	(50.5)	29	(26.6)	
Employed		43	(18.5)	2	(6.1)	10	(11.0)	31	(28.4)	
Unemployed		97	(41.6)	13	(39.4)	35	(38.5)	49	(45.0)	
Relationship status	226									< 0.000
No current partner		66	(29.2)	15	(46.9)	22	(24.7)	29	(27.6)	
Dating one partner/married		123	(54.4)	11	(34.4)	66	(74.2)	46	(43.8)	
Casual dating/multiple partners		37	(16.4)	6	(18.8)	1	(1.1)	30	(28.6)	
Mobile phone ownership and use	233									0.66
No		33	14.2	9	(4.7)	11	(12.1)	13	(11.9)	
Yes		200	85.8	24	(72.7)	80	(87.9)	96	(88.1)	
Mobile App usage	333									0.31
No		18	7.7	4	(12.1)	4	(4.4)	10	(8.4)	
>1		36	15.5	4	(12.1)	22	(24.2)	10	(16.8)	
+1		179	76.8	25	(75.8)	65	(71.4)	89	(81.7)	
Depressive symptoms	333									0.079
No		87	26.13	12	(36.4)	17	(18.7)	21	(19.3)	
Yes		246	73.87	21	(63.6)	74	(81.3)	88	(80.7)	
transactional sex engagement	229									0.000
No		150	(65.5)	24	(72.7)	75	(83.3)	51	(48.1)	
Yes		79	(34.5)	9	(27.3)	15	(16.7)	55	(51.9)	
Sexual Relationship Power	233	Mean = 30.33, SD = 6.40	Range = 11–44	Mean = 31.58, SD = 5.85	Range = 22–44	Mean = 33.36, SD = 6.22	Range = 22–44	Mean = 27.42, SD = 6.26	Range = 11–44	0.000
Adolescent sexual and reproductive health stigma	233	Mean = 13.68, SD = 4.08	Range = 1–19	Mean = 13.36, SD = 4.82	Range = 1–19	Mean = 14.30, SD = 3.35	Range = 4–19	Mean = 13.27, SD = 3.87	Range = 3–19	0.185
Food insecurity	233									0.850
No		71	(30.5)	9	(27.3)	27	(29.7)	35	(32.1)	
Yes		162	(65.5)	24	(72.7)	64	(70.3)	74	(67.9)	
Perceived Community safety	233									0.436
Not safe		99	(42.5)	11	(33.3)	38	(41.8)	50	(45.9)	
Safe		134	(57.5)	22	(66.7)	53	(58.2)	59	(54.1)	

Note: 1. *P*-values were calculated using ANOVA or Chi square test

*Types of violence included verbal, physical, and sexual violence

### Multinomial logistic regression of lifetime young adulthood violence experiences

Multinomial logistic regression (MLR) analyses were conducted to identify social ecological factors associated with experiencing young adulthood polyvictimization (> 16 years old). The reference category for the outcome variable was ‘no violence experiences’; each category was compared to this reference group.

#### Experiencing one type of young adulthood violence

In adjusted multinomial logistic regression analyses (Table 3), experiencing one type of violence vs. no violence was significantly associated with mobile app usage, whereby participants who used > 1 mobile app had higher odds of violence than their counterparts who did not use mobile apps (AOR 9.19, 95% CI = 2.09, 40.42). The odds of experiencing violence were higher for those reporting depressive symptoms vs no depression symptoms (AOR 3.17, 95% CI = 1.43, 4.15), reporting any childhood abuse (AOR 3.97, 95% CI = 1.70, 9.27) and childhood polyvictimization (AOR 3.65, 95% CI = 1.59, 8.39) vs. those with no childhood abuse. Increased sexual relationship power (AOR 1.04, 95% CI = 1.00, 1.08) and adolescent SRH stigma (AOR 1.15, 95% CI = 1.05, 1.28) scores were associated with experiencing one type of YAV vs. no violence.

#### Experiencing young adulthood polyvictimization

Adjusted multinomial logistic regression analyses (Table 3) indicate that the odds of experiencing young adulthood polyvictimization were higher for participants reporting depressive symptoms vs. no depressive symptoms (AOR 5.72 95% CI = 1.29, 25.38) and childhood polyvictimization (AOR 5.08 95% CI = 1.38, 18.64). Sexual relationship power (AOR 1.00 95% CI = 1.00, 1.19) and food insecurity (AOR 7.15 95% CI = 1.32, 38.89) were also associated with increased odds of young adulthood polyvictimization.

**Table 3:** Multinomial logistic regression findings of factors associated with violence among refugee/displaced adolescent girls and young women in Kampala, Uganda (N = 333)

Indicators	1 type of violence reported		+ 1 type of violence reported	
	Unadjusted OR (95 CI)	Adjusted OR (95 CI)	Unadjusted OR (95 CI)	Adjusted OR (95 CI)
*Socio-demographic factors*				
Age	1.13 (1.03, 1.24)**	1.07 (0.92, 1.24)	1.23 (1.06, 1.43)**	1.22 (0.97, 1.53)
Time in Uganda (> 1 year = 0)				
1–5 years	2.24 (0.83, 6.06)		1.81 (0.38, 8.53)	
<5 years	3.08 (0.99, 8.49)		1.15 (0.22, 6.02)	
Relationship status (single = 0)				
Dating one partner/married	1.67 (1.01, 2.75)*	1.18 (0.55, 2.55)	1.86 (0.78, 4.42)	1.24 (0.33, 4.61)
Casual dating/multiple partners	2.55 (1.14, 5.70)*	1.95 (0.63, 6.03)	3.14 (0.92, 10.76)	1.06 (0.17, 6.73)
Intrapersonal factors				
Mobile phone ownership and use (Reference: No phone = 1)	1.90 (1.14, 3.18)*	0.57 (0.20, 1.66)	1.10 (0.48, 2.51)	0.34 (0.07, 1.66)
Mobile App usage (No = 0)				
>1	1.37 (0.63, 2.96)	2.91 (0.57, 14.97)	0.99 (0.19, 5.15)	0.15 (0.01, 1.59)
+1	3.29 (1.80, 6.02)***	9.19 (2.09, 40.42)**	1.21 (0.52, 2.81)	1.06 (0.25, 4.52)
Depression (no symptoms = 0)	2.45 (1.45, 4.15)***	3.17 (1.43, 7.02)**	5.33 (1.55, 18.34)	5.72 (1.29, 25.38)*
*Interpersonal factors*				
Experienced victimization as a child (no = 0)				
I type of violence	2.02 (1.13, 3.63)**	3.97 (1.70, 9.27)***	0.89 (0.24, 1.60)**	1.08 (0.19, 6.09)
+1 type violence	2.41 (1.37, 4.24)**	3.65 (1.59, 8.39)**	4.29 (1.60, 11.45)**	5.08 (1.38, 18.64)*
Lifetime transactional sex (no = 0)	1.88 (1.08, 3.25)*	2.14 (0.95, 4.85)	5.96 (2.59, 13.71)***	2.39 (0.67, 8.48)
Sexual relationship power	1.04 (1.00, 1.08)*	1.06 (1.01, 1.12)*	1.04 (0.98, 1.09)	1.09 (1.00, 1.19)*
*Community factors*				
Adolescent SRH stigma	1.09 (1.03, 1.15)**	1.15 (1.05, 1.28)**	1.14 (1.02, 1.28)*	1.12 (0.96, 1.32)
Food insecurity (no = 0)	0.71 (0.44, 1.17)	0.98 (0.48, 2.07)	5.44 (1.24, 23.79)*	7.15 (1.32, 38.89)*
Perceived Community safety (unsafe = 0)	0.91 (0.58, 1.44)		0.65 (0.30, 1.41)	

Reference group consists of participants reporting no experience of violence after the age of 16. OR, odds ratio; CI, confidence interval; AGYW, adolescent girls and young women. Final adjusted model included: age, relationship status, mobile phone ownership, mobile app usage, depression, childhood violence, lifetime transactional sex, sexual relationship power, adolescent SRH stigma, food insecurity and community safety. *p = < 0.05; **p < 0.01; ***p < 0.000

### Multinomial logistic regression of recent intimate partner violence

In multinomial logistic regression analyses social ecological factors were examined in relation to experiencing IPV in the last 12 months (recent IPV) for persons who were currently dating/in relationship(s), or had dated in the past 12 months. Experiencing one type of IPV, and IPV polyvictimization, were compared to the reference group of no violence experiences.

#### Experiencing any IPV

Adjusted multinomial logistic regression analyses (Table 4) findings indicate that participants who were dating one partner or married had higher odds of experiencing recent IPV (AOR 3.37, 95% CI = 1.22, 9.31) than those who were currently single. Participants who had casual/multiple partners had lower odds of recent IPV compared to their currently single counterparts (AOR 0.08, 95% CI = 0.01, 0.78).

#### Experiencing IPV polyvictimization

In adjusted multinomial logistic regression analyses (Table 4), participants who owned and used mobile phones were more likely to experience recent IPV polyvictimization than their counterparts without mobile phones (AOR 3.65 95% CI = 1.15, 11.60). Depressive symptoms were also associated with higher odds of recent IPV polyvictimization (AOR 3.17 95% CI = 1.14, 8.83). Participants with higher sexual relationship power had lower odds of recent IPV polyvictimization (AOR 0.84 95% CI = 0.76, 0.92).

**Table 4:** Multinomial logistic regression of factors associated with recent IPV among refugee/displaced adolescent girls and young women in Kampala, Uganda (*N* = 233)

	1 type of violence experience		+ 1 type of violence experience	
Indicators	Unadjusted OR (95 CI)	Adjusted OR (95 CI)	Unadjusted OR (95 CI)	Adjusted OR (95 CI)
*Socio-demographic factors*				
Age	0.86 (0.73, 1.01)		0.99 (0.85, 1.17)	
Time in Uganda (>1 year = 0)				
1–5 years	5.00 (0.76, 32.77)		1.40 (0.34, 5.71)	
<5 years	3.90 (0.59, 25.70)		0.58 (0.14, 2.43)	
Relationship status (currently single = 0)				
Dating one partner/married	4.10 (1.64, 10.22)**	3.37 (1.22, 9.31)*	2.16 (0.87, 5.35)	1.25 (0.44, 3.40)
Casual dating/multiple partners	0.11 (0.01, 1.04)	0.08 (0.01, 0.78)*	2.59 (0.88, 7.58)	1.70 (0.47, 6.11)
Intrapersonal factors				
Mobile phone ownership and use (no = 1)	2.73 (1.01, 7.35)*	2.21 (0.72, 6.80)	2.77 (1.06, 7.24)*	3.65 (1.15, 11.60)*
Mobile App usage (no = 0)				
>1	5.49 (0.95, 31.59)		0.99 (0.19, 5.15)	
+1	2.60 (0.60, 11.20)		1.42 (0.41, 4.93)	
Depression (no symptoms = 0)	2.49 (1.03, 6.02)*	2.24 (0.81, 6.21)	2.39 (1.02, 5.63)*	3.17 (1.14, 8.83)*
*Interpersonal factors*				
Experienced victimization as a child (no = 0)				
I type of violence	1.49 (0.51, 4.29)		1.69 (0.59, 4.82)	
+1 type violence	1.23 (0.48, 3.12)		1.54 (0.61, 3.87)	
Lifetime transactional sex (no = 0)	0.53 (0.21, 1.37)	0.75 (0.25, 2.25)	2.88 (1.22, 6.77)*	2.20 (0.81, 5.94)
Sexual relationship power	1.05 (0.98, 1.12)	1.07 (0.98, 1.16)	0.87 (0.81, 0.94)***	0.84 (0.76, 0.92)***
Community factors				
Adolescent SRH stigma	1.06 (0.96, 1.69)		0.99 (0.91, 1.09)	
Food insecurity (no = 0)	0.89 (0.37, 2.16)		0.79 (0.33, 1.88)	
Perceived Community safety (not safe = 0)	0.69 (0. 30, 1.61)		0.60 (0.2, 1.33)	

Reference group consists of participants reporting no experience of intimate partner violence in the last 12 months. OR, odds ratio; CI, confidence interval; AGYW, adolescent girls and young women. Final adjusted model included: relationship status, mobile phone ownership, depression, lifetime transactional sex, sexual relationship power, adolescent SRH stigma. *p = < 0.05; **p < 0.01; ***p < 0.000

## Discussion

This community-based study found alarming levels of violence in young adulthood—over half (53.8%) of participants reported experiencing any violence after the age of 16—among refugee AGYW in Kampala’s urban settlements. Most participants had been in relationships in the past year, with the overwhelming majority of these (85.8%) reporting IPV in that timeframe. Recent IPV was associated with currently being in a relationship, mobile phone ownership/use and mobile app use, and lower sexual relationship power. Findings can inform practice, policy and intervention development tailored for urban refugee and displaced AGYW in Kampala.

Our finding that 54.7% of urban refugee and displaced AGYW experienced any young adulthood violence is consistent with estimates of violence experienced by non-refugee samples of AGYW living in Kampala’s informal settlements (53.6%) [22], signalling a larger contextual issue of violence in informal settlements that warrants urgent attention. Neighbourhood effects in slums created by shared physical and social environments may position AGYW as particularly vulnerable to violence, while simultaneously presenting the opportunity for violence interventions to realize economies of scale in slums due to these neighbourhood effects [52]. The prevalence of violence in this study is comparable with estimates from a multi-country study with conflict-affected adolescent girls in the DRC and Ethiopia (51.6%) [20] and among Congolese refugee women in Rwanda (49%) [53]. Notably the violence reported in this study is higher than among adolescents and young people in at large in Uganda (33.3%) [18]. Taken together, there is an urgent need for community level violence prevention initiatives that address poverty, social exclusion and disenfranchisement in both urban refugee and urban slum communities in Kampala. Among urban refugees, these neighbourhood effects may be the most salient factors shaping violence exposure. Study implications thus point to the need for SGBV integration within larger slum health interventions to tackle violence among urban refugees—and this would entail working with host communities. This may contrast with strategies within refugee settlements that may not be as integrated with host communities.

Recent IPV was highly concerning: most (85.9%) urban refugee AGYW who were in an intimate partnership in the last year reported at least one IPV incident in the last 12 months, higher than IPV estimates among women in sub-Saharan Africa (37%) [2], post-conflict Northern Uganda (52%) [14], conflict-affected South Sudan (43.1%) [15] and conflicted-affected DRC (68%) [13]. We did not find comparable studies on IPV with settlement-based refugee and displaced AGYW in Uganda. The recent IPV estimates in our study may have been higher as we included ‘control’ as a form of violence, whereas other studies only considered sexual and physical violence. Our estimates of IPV polyvictimization—experiencing more than one type of IPV—is also higher among our sample (46.8%) compared to the national average (35%) [18]. There is need for the expansion of the Domestic Violence Act of 2010 to respond to violence in cohabiting or dating relationships. Addressing root causes and sequalae of recent IPV among urban refugee AGYW in Kampala should be a public health priority.

Social ecological factors associated with experiencing young adulthood violence included *intrapersonal* (depression, mobile app usage), *interpersonal* (childhood abuse, lifetime transactional sex, sexual relationship power), and *community* (adolescent SRH stigma, food insecurity) level factors. Consistent with prior work [5] in humanitarian contexts, we found that adversities such as childhood abuse increased exposure to future violence. Studies have linked experiences of household violence, including childhood abuse, to IPV, especially among conflict-affected populations [26]. Our findings that childhood abuse was associated with young adulthood violence, including polyvictimization, contributes to this evidence base with conflict-affected populations. During humanitarian situations children are more susceptible to violence, that in turn may have long lasting psychosocial effects that elevate risks for future victimization [9]. Violence occurs before, during and after conflict. For instance, a study in Cote d’Ivoire found that IPV doubled in post-conflict periods [54].

We also found that food insecurity was associated with increased exposure to young adulthood polyvictimization among urban refugee and displaced AGYW. This corroborates research that identifies poverty and its sequelae—including men’s frustrations with attempting to realize financial security for themselves and their families—with increased violence towards women in post-conflict Sierra Leone [55], Liberia [55], Colombia [31] and post-earthquake Haiti [27]. The high violence targeting both refugee and non-refugee AGYW in Kampala’s informal settlements calls for slum specific interventions to foster economic stability, upgrade physical infrastructure, and health and educational programs [52, 56]. While we organized findings following Rubenstein and Stark’s application of the social ecological model to violence in humanitarian emergencies [9], our findings also align with Heise’s ecological framework of violence that conceptualizes personal history (i.e. childhood abuse), microsystem (i.e. sexual relationship power), exosystem (i.e. food insecurity) and macrosystem (i.e. SRH stigma) factors related to violence against women [57].

We also found an association between depressive symptoms and experiencing young adulthood violence. While our cross-sectional survey cannot infer causality, there is likely a reciprocal relationship between violence and depression. A systematic review of cohort studies among women reported that IPV is associated with ensuing depression, and experiencing depression is associated with subsequent IPV [36]. Depressive symptoms may include low self-esteem, low self-efficacy, social isolation and hopelessness, these symptoms may constrain persons from achieving resources and services needed to leave abusive relationships [38]. There is a need for longitudinal research with urban refugee and displaced AGYW to better understand temporal associations between depression and IPV.

Findings raise questions regarding the role of mobile phones and apps and vulnerability to violence among urban refugee and displaced AGYW. For instance, we found an association between using multiple mobile apps and young adulthood violence, and between mobile phone ownership and recent IPV. This corroborates literature that explores the ways that technology can facilitate SGBV toward women and girls [29], and the need to address larger social, cultural, economic and political contexts that (re) produce gender inequitable norms that underpin SGBV. Much attention has focused on the potential of mobile phones and apps as empowering and health promoting tools [58], particularly for refugees [41]. The World Health Organization’s [58] recent guidelines encouraged countries to harness the power of digital technologies to improve health outcomes. Technology-facilitated gender-based violence [42] is understudied in humanitarian contexts. Future research with urban refugee AGYW can explore both the potential for violence reduction, and possible risk for harm, associated with mobile technologies [44, 45].

Sexual relationship power—control over practices and decision making in relationships— was associated with increased exposure to young adulthood violence, but acted a protective factor for recent IPV. Without longitudinal data it is difficult to understand changes over time in refugee and displaced AGYW’s sexual relationship power. It is plausible that when refugee and displaced AGYW migrate to urban slums, they may experience increased social and/or economic independence and changed gender roles in urban settings compared to refugee camps/settlements and conflict situations. Consequences of increased sexual relationship power could include men using violence against women to regain dominance [31, 55], (re) producing broader structures of inequitable gender norms in host communities.

This may also help to understand the associations we identify between adolescent SRH stigma and young adulthood violence. While underexplored in relation to violence, adolescent SRH stigma is associated with less uptake of contraception [59]— an indicator of sexual decision making, a core element of sexual relationship power. It is also possible that refugee and displaced young women could experience violence as a backlash for accessing SRH services. Our finding that sexual relationship power was associated with lower odds of recent IPV reflects research in the U. S [60] that posits that relationship control can be protective. Women with greater control over their own and their intimate partner’s behaviours and sexual decisions may be less likely to accept IPV, and/or may have the ability to access resources and support to end unhealthy relationships. It may also be a reciprocal process, whereby persons experiencing IPV may feel lower sexual relationship power. Gender power asymmetries in relationships require further attention with conflict-affected AGYW. How gender norms and the construction of masculinities are impacted in conflict settings, particularly in urban areas, is a critical area for future research. Our findings also point to the need for gender-transformative interventions tailored for urban refugee contexts [61], harnessing digital technology and social media to reach both women and men of varied ages across informal settlements.

Intervention strategies for SGBV reduction tailored for urban refugee and displaced AGYW can consider both risk and protective factors across the socio-ecological framework for sustained impact. At the individual level, to address depressive symptoms, trauma-informed care can recognize and respond to trauma, promote safety and healing, and provide tools to leave IPV contexts [62]. In areas with few trained mental health providers, refugees and displaced persons can be trained to provide trauma-informed programs with violence survivors. Lay mental health providers can improve access and use of mental health services for marginalized populations [63], including conflict affected persons. Digital technologies can be used to train, monitor and supervise lay mental health providers [58, 63]. Community wide interventions can tackle inequitable gender norms rooted in cultural, social and economic systems. Structural interventions focused on economic empowerment for conflict-affected persons have the potential to address food insecurity as well as SGBV. Though there is scant evidence on the effectiveness of GBV prevention interventions in humanitarian settings, particularly for youth, [64], family strengthening interventions may improve wellbeing among refugee households [65].

There are study limitations. First, the cross-sectional design precludes inferring causality, therefore we restricted independent variable to relationships for which temporality could be reasonably inferred. There is need for future studies to use longitudinal methods to explore causal pathways of violence toward forcibly displaced AGYW. Second, although we considered five informal settlements of Kampala, Uganda, there is need for future studies to explore the geographic and social distribution of different types of violence across communities to inform targeted community interventions and policies. Third, our study did not explore the identity of the perpetrators, or the places where AGYW experienced violence. This was part of a parent study on HIV prevention, hence we used brief assessments of violence. The use of single measures of violence means that we might not have captured the extent and levels of violence experienced by participants. Future studies should use comprehensive, standardized, multi-dimensional instruments to assess violence. Fourth, our study did not explore the overlap between YAV and IPV. There is need for future studies to asses this, for instance using advanced statistical methods such as latent class analysis [66], to identify patterns of violence. Fifth, we used a non-probability sample, which means that we cannot generalize study findings. Research is needed to establish prevalence. Sixth, although our study found that mobile phone and mobile app usage were associated with violence experiences, we did not explore technology facilitated SGBV. Future studies of technology facilitated SGBV among urban refugees and displaced persons can inform technology-based interventions. Finally, due to small sub-groups we were not able to explore differences by violence across more marginalized AGYW, such as those with disabilities. This is an important area for research. Despite these limitations, our study broadens the evidence base on SGBV among urban refugee and displaced AGYW.

## Conclusion

This study provides information regarding the prevalence and correlates of young adulthood violence and recent IPV among urban refugee and displaced AGYW in Kampala’s informal settlements. Findings highlight the need for comprehensive interventions that address social, economic and cultural gender-based inequities that affect both urban forcibly displaced persons and host communities. There is urgent need for studies to explore digital health technology use among urban refugee AGYW and its associations with risk for SGBV [42] as well as its potential use in SGBV prevention [67]. New WHO guidelines on self-care interventions for sexual and reproductive health and rights include digital technologies and platforms as potential self-care interventions to improve autonomy and agency among AGYW [68]. This approach conceptualizes decision making across all facets of life—including sexuality and relationships—within key principles of agency, gender equality, human rights and the lifecourse [69]. The potential for technologies to advance SRH self-care and rights among AGYW in humanitarian contexts is understudied and a key area for future research [70]. Addressing polyvictimization directly, as well as the social and structural contexts within which violence targeting AGYW is produced in families, communities and intimate relationships, is essential for advancing health, wellbeing and human rights among urban refugee and displaced AGYW.

## Data Availability

The datasets generated and/or analysed during the current study are not publicly available due to research ethics board restrictions but are available from the corresponding author on reasonable request and on attaining research ethics board amendments from the University of Toronto and Ugandan Ministry of Health.
